# Acute Septic Arthritis of the Knee Caused by *Kingella kingae* in a 5-Year-Old Cameroonian Boy

**DOI:** 10.3389/fped.2017.00230

**Published:** 2017-11-06

**Authors:** Nawal El Houmami, Dimitri Ceroni, Karine Codjo Seignon, Jean-Christophe Pons, Cédric Lambert, Guillaume André Durand, Philippe Minodier, Léopold Lamah, Philippe Bidet, Jacques Schrenzel, Didier Raoult, Pierre-Edouard Fournier

**Affiliations:** ^1^Research Unit on Infectious and Emerging Tropical Diseases (URMITE), UM63, CNRS 7278, IRD 198, INSERM 1095, Aix-Marseille Université, IHU Méditerranée Infection, Marseille, France; ^2^Département de l’enfant et de l’adolescent, Hôpital des Enfants, Hôpitaux Universitaires de Genève (HUG), Geneva, Switzerland; ^3^Department of Pediatrics, Dracénie Hospital, Draguignan, France; ^4^Department of Pediatric Emergency Medicine, North Hospital, Aix-Marseille Université, Marseille, France; ^5^Department of Orthopedics and Traumatology, Donka University Hospital, University of Conakry Gamal Abdel Nasser, Conakry, Guinea; ^6^Laboratoire de Microbiologie, Hôpital Robert Debré, Assistance Publique – Hôpitaux de Paris, Université Paris Diderot, Sorbonne Paris Cité, INSERM, IAME, UMR 1137, Paris, France; ^7^Bacteriology and Genomic Research Laboratories, Geneva University Hospitals (HUG) and Geneva University, Geneva, Switzerland

**Keywords:** *Kingella kingae*, pediatrics, arthritis, infectious, multilocus sequence typing, Africa South of the Sahara

## Abstract

*Kingella kingae* is an important cause of invasive infections in young children from Western countries. Although increasing reports indicate that this organism is the leading agent of bone and joint infections in early childhood, data on *K. kingae* infections from resource-limited settings are scarce, and none has yet been reported in Africa. We herein report the diagnostic and epidemiological investigations of the first case of *K. kingae* arthritis identified in a child from sub-Saharan Africa. A 5-year-old Cameroonian boy presented with a sudden painful limp which appeared in the course of a mild rhinopharyngitis. He lived in Cameroon where he had been vaccinated with BCG at birth and moved to France for holidays 4 days before consultation. There was no history of trauma and he did not have any underlying medical condition. Upon admission, he had a temperature of 36.7°C, and clinical examination revealed right-sided knee tenderness and effusion that was confirmed by ultrasound imaging. Laboratory results showed a white blood cell count of 5,700 cells/mm^3^, C-reactive protein level of 174 mg/L, and platelet count of 495,000 cells/mm^3^. He underwent an arthrocentesis and was immediately given intravenous amoxicillin-clavulanate. Conventional cultures from blood samples and synovial fluids were negative. Polymerase chain reaction (PCR) assay targeting the broad-range 16S rRNA gene and real-time quantitative PCR assays targeting *Mycobacterium* species were negative. Surprisingly, real-time PCR assays targeting the *cpn60, rtxA*, and *rtxB* genes of *K. kingae* were positive. Multicolor fluorescence *in situ* hybridization specific for *K. kingae* identified the presence of numerous coccobacilli located within the synovial fluid. Finally, multilocus sequence typing analysis performed on deoxyribonucleic acid directly extracted from joint fluid disclosed a novel *K. kingae* sequence-type complex. This case report demonstrates that *K. kingae* may be considered as a potential cause of septic arthritis in children living in sub-Saharan Africa, and hence the burden of *K. kingae* infection may be not limited to the Western countries. Further studies are required to determine the prevalence of *K. kingae* infection and carriage in Africa.

## Background

*Kingella kingae* is an emerging pathogen recognized as the primary etiology of bone and joint infections in young children from Western countries (1, 2). Asymptomatically harbored in the oropharynx of children aged 6–48 months, the prevalence of *K. kingae* oropharyngeal carriage ranges from 8 to 23% from studies carried out in Israel, Switzerland, and New Zealand (3–6). Because this Gram-negative bacterium is usually responsible for a mild to moderate inflammatory response, and its detection is notoriously difficult by conventional culture, diagnosis of *K. kingae* infection requires a high index of suspicion and the use of adequate detection methods such as real-time quantitative polymerase chain reaction (qPCR) assays (6, 7). These molecular diagnostic tools exhibit higher sensitivity compared with culture methods, shorten the time of detection from days to a few hours, and enable the identification of the organism among healthy carriers (4–6).

Large-scale epidemiological studies based on multilocus sequence typing (MLST) analysis of *K. kingae* showed that dominant clones belonging to sequence-type complexes 6 (STc-6), -14, -23, and -25 accounted for 72% of strains disseminated worldwide, mainly in the USA, Europe, and Israel, with ST-14 and ST-25 being positively associated with osteoarticular infections (8). To date, *K. kingae* infection and carriage have been studied in Israel, Europe, North and South America, Australia, New Zealand, and Japan (5, 8–10), but none have yet been reported in Africa. We herein report the diagnostic and epidemiological investigations of *K. kingae* arthritis in a young, previously healthy child from Cameroon, and we discuss the clinical implications of these findings.

## Case Presentation

On 11 July 2016, a 5-year-old Cameroonian boy was admitted to the emergency department at the Dracénie Hospital in the region Provences-Alpes-Côte d’Azur, France, due to a painful limp that appeared in the morning. He lived in Cameroon where he had been vaccinated with BCG at birth, and moved to Southeastern France for holidays 4 days before consultation. A mild rhinopharyngitis had occurred the previous week, but as the symptoms were mild, no treatment had been undertaken. There was no history of trauma, and he did not have any underlying medical condition. Upon admission to hospital, the child had a temperature of 36.7°C and refused to walk. Clinical examination revealed right-sided knee tenderness and effusion. Neither skin rash nor oral ulcerations were noted. Laboratory results showed an elevated C-reactive protein (CRP) level at 174 mg/L, with normal white blood cell count of 5,700 cells/mm^3^ and platelet count of 495,000 cells/mm^3^. Ultrasound imaging confirmed effusion of the right knee, whereas conventional radiograph showed no significant abnormality. The child underwent an arthrocentesis, and mildly opaque and yellowish liquid was extracted, suggesting a septic arthritis of the right knee. Consequently, the child was immediately given intravenous amoxicillin-clavulanate 100 mg/kg three doses daily during 3 days.

## Description of Laboratory Investigations and Diagnostic Tests

**Figure 1:**
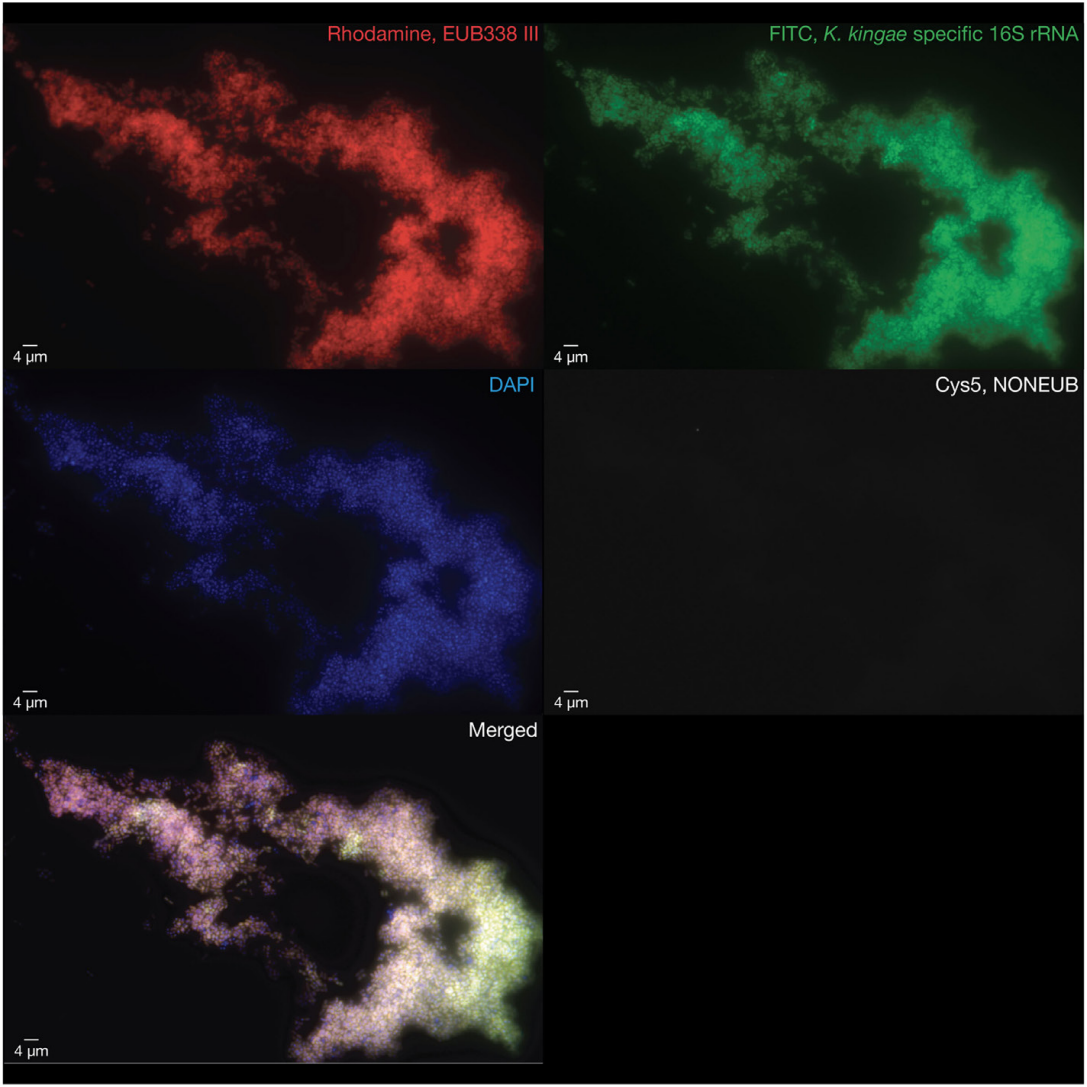
Multicolor fluorescence *in situ* hybridization assays were performed on the pathogenic synovial fluid after this latter was formalin fixed and paraffin embedded. Large numbers of viable coccobacilli representing the causative *Kingella kingae* strains are visualized in red using a rhodamine-labeled probe targeting a consensus sequence of the bacterial 16S rRNA gene (EUB388 III, top left), in green using an FITC-labeled probe targeting the *K. kingae*-specific V1 region of the 16S rRNA gene (top right), and in blue using a DAPI probe to label deoxyribonucleic acid (middle left). An internal negative control was performed by using a bacterial non EUB388 probe (middle right). The merged image was obtained summing the four abovementioned images (bottom left).

Because conventional cultures applied for Gram-positive, Gram-negative, mycobacterial species, and fungi from the joint fluid and blood samples were negative, joint specimens were sent in dry ice to the molecular diagnosis laboratory of the URMITE unit in Marseille, where bacterial deoxyribonucleic acid (DNA) was extracted directly from the joint fluid. Polymerase chain reaction (PCR) assay targeting the broad-range bacterial 16S rRNA gene (11) and qPCR assays targeting both *Mycobacterium* species and *Mycobacterium tuberculosis* complex (12) were negative. Given the age of the patient, *K. kingae* was also sought by using specific qPCR assay targeting the *K. kingae cpn60* (*groEL*) gene (11). Surprisingly, this specific *K. kingae* assay was positive, as well as qPCR assays targeting the *Kingella*-specific *rtxA* and *rtxB* genes (7, 13), thus confirming the diagnosis of septic arthritis caused by *K. kingae*. The organism was also identified by multicolor fluorescence *in situ* hybridization specific for *K. kingae* (Figures S1 and S2 in Supplementary Material), which revealed the presence of large numbers of viable coccobacilli located within the synovial fluid (Figure 1). Cardiac investigations ruled out endocarditis. A switch to oral amoxicillin-clavulanate 100 mg/kg three doses daily was then undertaken on 15 July 2016 and was planned for a total duration of 2 weeks. Despite these recommendations, the treatment was continued for another 2 months in Cameroon. During the final follow-up 3 months postoperatively, clinical examination revealed a normal knee status with a normal range of motion.

Thereafter, MLST studies using a modified protocol specific for *K. kingae* was performed on bacterial DNA extracted directly from the joint fluid as previously described (14). Five alleles were unambiguously identified, namely, *adk-2, aroE-2, cpn60-2, zwf-13*, and *recA-2*. Unexpectedly, 14 single nucleotide variants of the *abcZ* allele were identified from nucleotides 6–447 (Figure S3 in Supplementary Material; Table 1). To estimate the between-strain relatedness and define an MLST scheme for *K. kingae*, a different allele number was given to each distinct sequence within a locus, and a distinct sequence-type (ST) number was attributed to each distinct allele combination (15). *K. kingae* isolates were then grouped into ST-complexes (STcs) if they differed at no more than one locus from at least one other member of the group. Among the 70 STs of *K. kingae* that are documented in the multilocus sequence database (MLST) of the Institut Pasteur database (http://bigsdb.pasteur.fr/perl/bigsdb/bigsdb.pl?db=pubmlst_kingella_seqdef_public&page=downloadProfiles&scheme_id=1), ST-26, which belongs to the highly invasive STc-25, was the closest ST by sharing four alleles, namely, *adk-2, cpn60-2, gdh/zwf-13*, and *recA-2* with the causative strains that were herein identified (Table 2). Although analysis of the combination produced by the five unambiguous alleles indicated that the causative *K. kingae* strains belongs to a novel ST, the presence of multiple *abcZ* alleles does not allow to precisely define it. Moreover, in the MLST scheme of *K. kingae*, founder genotypes of STcs were defined as the ST of the STc with the highest number of neighboring STs [(15), Table 3]. Consequently, although analysis of the combination produced by the five unambiguous alleles indicated that the causative *K. kingae* strains belong also to a novel STc, no specific denomination is yet possible. Moreover, since each of these housekeeping genes is present in one copy in the whole genome of *K. kingae*, these findings suggested co-infection by strains belonging to distinct STs.

**Table 1:** Distance matrices of the *Kingella kingae* abcZ allele corresponding to the MAFFT alignment displayed in Figure S3 in Supplementary Material.

	abcZ-F_1574363	abcZ-R_1574363	abcZ_5	abcZ_1	abcZ_2	abcZ_3	abcZ_4	abcZ_6	abcZ_7	abcZ_8	abcZ_9	abcZ_10	abcZ_11	abcZ_12	abcZ_13	abcZ_14	abcZ_15	abcZ_16	abcZ_17	abcZ_18	abcZ_19	abcZ_20	abcZ_21
abcZ-F_1574363		98.53	98.45	98.23	96.69	97.35	96.03	88.3	96.69	96.8	83.89	95.81	97.68	96.8	96.58	97.68	98.01	83.66	96.91	88.08	98.01	96.47	88.08
abcZ-R_1574363	98.53		98.53	98.3	96.72	97.4	96.27	88.12	96.72	96.83	84.5	96.04	97.74	96.83	96.61	97.74	98.08	84.28	96.95	87.9	98.08	96.49	87.9
abcZ_5	98.45	98.53		96.69	95.58	96.03	95.81	87.86	95.36	95.81	83.22	95.58	96.47	95.58	96.25	96.47	96.47	83	95.81	87.64	96.47	95.36	87.64
abcZ_1	98.23	98.3	96.69		97.57	98.9	96.47	88.96	97.79	97.57	84.55	96.25	98.68	97.79	97.13	99.12	99.34	84.33	97.79	88.74	99.78	97.79	88.74
abcZ_2	96.69	96.72	95.58	97.57		96.47	96.69	90.29	99.78	96.91	84.11	96.47	97.57	98.01	96.91	97.57	97.35	83.89	97.13	90.07	97.35	99.78	90.07
abcZ_3	97.35	97.4	96.03	98.9	96.47		95.81	87.86	96.69	96.47	83.89	95.58	98.01	97.13	96.03	98.45	98.68	83.66	96.69	87.64	98.68	96.69	87.64
abcZ_4	96.03	96.27	95.81	96.47	96.69	95.81		88.96	96.47	94.92	84.33	99.78	96.03	97.35	97.13	96.47	95.81	84.33	95.14	88.74	96.25	96.91	88.74
abcZ_6	88.3	88.12	87.86	88.96	90.29	87.86	88.96		90.07	87.86	91.39	88.96	88.52	89.4	88.3	88.96	88.3	91.17	88.3	99.78	88.74	90.51	99.78
abcZ_7	96.69	96.72	95.36	97.79	99.78	96.69	96.47	90.07		97.13	84.33	96.25	97.79	98.23	96.69	97.79	97.57	84.11	97.35	89.85	97.57	99.56	89.85
abcZ_8	96.8	96.83	95.81	97.57	96.91	96.47	94.92	87.86	97.13		84.77	94.7	97.13	97.13	97.35	97.13	97.35	84.55	99.34	87.64	97.35	96.69	87.64
abcZ_9	83.89	84.5	83.22	84.55	84.11	83.89	84.33	91.39	84.33	84.77		84.33	84.33	84.55	83.22	84.11	84.11	99.78	84.77	91.39	84.33	84.11	91.17
abcZ_10	95.81	96.04	95.58	96.25	96.47	95.58	99.78	88.96	96.25	94.7	84.33		95.81	97.13	96.91	96.25	95.58	84.33	94.92	88.74	96.03	96.69	88.74
abcZ_11	97.68	97.74	96.47	98.68	97.57	98.01	96.03	88.52	97.79	97.13	84.33	95.81		98.23	96.25	99.56	98.45	84.11	96.91	88.3	98.45	97.35	88.3
abcZ_12	96.8	96.83	95.58	97.79	98.01	97.13	97.35	89.4	98.23	97.13	84.55	97.13	98.23		97.13	98.23	97.57	84.33	96.91	89.18	97.57	97.79	89.18
abcZ_13	96.58	96.61	96.25	97.13	96.91	96.03	97.13	88.3	96.69	97.35	83.22	96.91	96.25	97.13		96.69	96.47	83	96.69	88.08	96.91	97.13	88.08
abcZ_14	97.68	97.74	96.47	99.12	97.57	98.45	96.47	88.96	97.79	97.13	84.11	96.25	99.56	98.23	96.69		98.45	83.89	96.91	88.74	98.9	97.79	88.74
abcZ_15	98.01	98.08	96.47	99.34	97.35	98.68	95.81	88.3	97.57	97.35	84.11	95.58	98.45	97.57	96.47	98.45		83.89	97.57	88.08	99.12	97.13	88.08
abcZ_16	83.66	84.28	83	84.33	83.89	83.66	84.33	91.17	84.11	84.55	99.78	84.33	84.11	84.33	83	83.89	83.89		84.55	91.17	84.11	83.89	90.95
abcZ_17	96.91	96.95	95.81	97.79	97.13	96.69	95.14	88.3	97.35	99.34	84.77	94.92	96.91	96.91	96.69	96.91	97.57	84.55		88.08	97.57	96.91	88.08
abcZ_18	88.08	87.9	87.64	88.74	90.07	87.64	88.74	99.78	89.85	87.64	91.39	88.74	88.3	89.18	88.08	88.74	88.08	91.17	88.08		88.52	90.29	99.56
abcZ_19	98.01	98.08	96.47	99.78	97.35	98.68	96.25	88.74	97.57	97.35	84.33	96.03	98.45	97.57	96.91	98.9	99.12	84.11	97.57	88.52		97.57	88.52
abcZ_20	96.47	96.49	95.36	97.79	99.78	96.69	96.91	90.51	99.56	96.69	84.11	96.69	97.35	97.79	97.13	97.79	97.13	83.89	96.91	90.29	97.57		90.29
abcZ_21	88.08	87.9	87.64	88.74	90.07	87.64	88.74	99.78	89.85	87.64	91.17	88.74	88.3	89.18	88.08	88.74	88.08	90.95	88.08	99.56	88.52	90.29	

*The 453 nucleotides composing the nucleotide sequence of the *abcZ* allele sequenced from the specimen no. 1574363 (abcZ-F_1574363 and abcZ-R_1574363) range between 98.45% with *abcZ_5* and 83.66% with *abcZ_16*. This table was performed by using Geneious 10.2.3 (Biomatters)*.

*The degree of allele similarity is expressed by a Blue scale scheme code, with the most divergent allelles being displayed in dark blue and the most similar in light blue*.

**Table 2:** Among the 70 sequence types (STs) of *Kingella kingae* that are documented in the multilocus sequence database (MLST) of the Institut Pasteur database (http://bigsdb.pasteur.fr/perl/bigsdb/bigsdb.pl?db=pubmlst_kingella_seqdef_public&page=downloadProfiles&scheme_id=1), no. 1574363 shares four alleles, namely, *adk-2, cpn60-2, gdh/zwf-13*, and *recA-2*, with ST-26, which belongs to the ST complex (STc)-25; ST-26 is therefore the closest ST to the causative strains no. 1574363.

Reference	STc	ST	*abcZ*	*adk*	*aroE*	*cpn60*	*gdh/zwf*	*recA*
No. 1574363	NA	NA	NA	2	2	2	13	2
ST-26	25	26	7	2	6	2	13	2

*NA indicates data not available*.

**Table 3:** Multilocus sequence typing (MLST) scheme of *Kingella kingae* shows the combination of the six alleles used to define the sequence types (STs) and sequence-type complexes (STcs) of *K. kingae*.

STc	ST	*abcZ*	*adk*	*aroE*	*cpn60*	*gdh*/*zwf*	*recA*
1	1	1	1	1	1	1	1
1	2	1	1	1	1	1	3
3	3	14	9	14	1	7	4
NA	4	3	3	9	3	7	3
NA	5	4	2	9	3	7	3
6	6	5	2	4	5	5	1
6	7	5	2	13	5	5	1
NA	8	11	2	3	7	7	2
NA	9	11	2	4	3	4	3
NA	10	1	8	3	6	1	3
11	11	13	2	4	2	8	6
11	12	15	2	4	2	8	6
NA	13	3	3	3	3	10	4
14	14	3	3	3	3	3	3
14	15	3	3	3	3	12	3
14	16	3	3	12	3	3	3
14	17	3	2	3	3	3	3
14	18	8	3	3	3	3	3
NA	19	4	4	4	4	1	3
NA	20	4	2	3	4	1	3
23	21	10	2	2	2	2	2
23	22	4	2	2	2	2	2
23	23	2	2	2	2	2	2
23	24	2	2	8	2	2	2
25	25	7	2	6	2	2	2
25	26	7	2	6	2	13	2
NA	27	12	6	10	3	9	2
29	28	9	2	7	3	4	3
29	29	9	2	4	3	4	3
NA	30	16	10	7	3	4	3
NA	31	6	1	4	3	1	5
32	32	6	5	5	3	6	5
NA	33	6	7	11	3	11	5
NA	34	6	7	11	3	2	5
35	35	1	8	15	8	1	3
NA	36	1	11	15	8	1	3
NA	37	3	3	3	3	2	3
NA	38	6	7	11	3	2	2
3	39	14	9	14	1	7	10
NA	40	9	2	7	10	4	3
14	41	3	3	9	3	3	3
14	42	3	3	3	3	14	3
NA	43	3	2	3	3	15	11
23	44	4	2	2	2	2	7
6	45	5	2	4	5	5	9
6	46	5	2	6	5	5	1
NA	47	6	7	11	3	17	12
NA	48	1	1	17	2	16	2
NA	49	1	1	17	9	16	2
NA	50	17	1	1	11	1	8
NA	51	4	6	9	4	1	3
35	52	1	8	3	8	1	3
NA	53	18	2	4	2	9	3
NA	54	3	2	3	3	1	11
23	55	19	2	2	2	2	2
23	56	20	2	6	2	2	2
14	57	3	3	3	3	18	3
35	58	1	8	15	8	19	3
6	59	5	2	18	5	5	1
14	60	8	3	3	3	3	13
6	61	5	2	4	5	20	1
23	62	2	2	6	2	2	2
NA	63	14	2	19	1	7	10
NA	64	3	2	16	3	3	3
NA	65	21	7	11	3	17	5
32	66	6	5	10	3	6	5
NA	67	5	2	3	2	2	2
NA	68	5	2	6	11	9	1
NA	69	1	2	6	2	1	14
23	70	2	2	20	2	2	2
NA	NA	NA	2	2	2	13	2

*NA indicates data not yet available*.

*In the present case, MLST sequencing data from the joint fluid specimen no. 1574363 indicated that the *K. kingae* causative strains shared four alleles with ST-26/STc-25, namely, *adk-2, cpn60-2, gdh/zwf-13*, and *recA-2*. Therefore, ST-26/STc-25 is the closest ST with the *K. kingae* strains that were identified in the synovial fluid no. 1574363 (boxes designed on blue background in the bottom row of the table). Please note that among the 70 STs identified, 31 *K. kingae* isolates have not yet an STc determined (boxes designed on orange background)*.

## Discussion

To the best of our knowledge, we herein report the first case of laboratory-confirmed invasive infection due to *K. kingae* in a child living in Africa. Little is known of the epidemiology of pediatric bone and joint infections in the African continent; however, it is largely recognized that *Staphylococcus aureus* is the most common pathogen cultured in children with septic arthritis in resource-limited settings (10, 16). Nevertheless, septic arthritis caused by *S. aureus* affects most frequently older children and is more prone to result in a higher systemic inflammatory response when compared with *K. kingae* infections, and the organism is recovered without difficulty by culture of blood and synovial fluid aspirates (10, 16, 17). Although *K. kingae* arthritis is characterized by normal to moderate increase in inflammatory markers, we point out that the patient had a markedly elevated CRP level upon admission, consistent with invasive infection caused by *K. kingae* of at least several days duration. Despite this, *K. kingae* infection was highly suspected because this pathogen is recognized as the first cause of culture-negative, acute septic arthritis in young children and affects most commonly the knee (1). In addition, it was also demonstrated that viral respiratory infections may play a role in the pathogenesis of the disease by damaging the mucosal lining of the oral cavity, thus facilitating the spread of the organism from blood to distant anatomic sites (2).

The detection of *K. kingae* is currently improved by sensitive culture methods such as Bactec/Alert vials, and above all by specific qPCR assays (2, 7). However, these diagnostic methods are costly and not yet available in developing countries in which diagnostic resources such as blood culture or molecular assays are scarce, and hence the recognition of *K. kingae* as a possible cause of acute septic arthritis in pediatrics is particularly challenging. In low-income, high-burden settings of tuberculosis, antibiotics with appropriate coverage against *S. aureus* and classical pyogenic bacteria may be frequently administered without any cultures and in the case of non-response to antibiotic treatment, antituberculous drugs may be given empirically for several weeks or months.

Although the child presented with an arthritis caused by *K. kingae* 4 days after arrival in Southeastern France, we highlight that *K. kingae* infection usually develop in several days to weeks following oropharyngeal *K. kingae* carriage and viral infections (18). Moreover, MLST analysis of invasive *K. kingae* strains from Southeastern France in 2016 demonstrated that strains causing osteoarticular infections belonged to ST-6 and ST-25 in the large majority of cases (14). Taken together with the novel *K. kingae* STc herein described, these findings are consistent with the fact that the child acquired causative *K. kingae* strains in Cameroon.

Notably, in an unpublished pilot study, *K. kingae* has been identified in the oropharynx of young children from Western Africa. This study was carried out at the Donka University Hospital in Conakry, Guinea, from 2012 to 2013 (Ceroni and Lamah, unpublished data). To define the prevalence rate of oropharyngeal *K. kingae* carriage, 45 healthy children aged from 6 to 48 months were enrolled in this study. Children admitted for either elective surgery or attending the orthopedic outpatient clinic or visiting the emergency department for non-infectious disease were included, whereas those presenting an invasive infectious disease, or administration of antimicrobial drugs the two preceding months were excluded. Recent travel abroad was not reported in any child. Oropharyngeal specimens were obtained by rubbing a cotton swab on the child’s tonsils, which were subsequently tested by molecular assays described earlier (13). Three children tested positive for *K. kingae*, thus indicating a prevalence rate of 6.7%, which is roughly similar to that observed in Europe (4). Despite the small size of this pilot study, these preliminary results provide evidence that *K. kingae* is circulating in Western Africa as well, and as a result, *K. kingae* might be considered as a potential pathogen responsible for septic arthritis in young children living in this geographical area. Early microbiologically proven diagnosis of *K. kingae* infection would enable to provide appropriate antibiotic therapy by amoxicillin, or amoxicillin-clavulanate, and to drastically reduce the total duration of treatment to a few days or weeks (2, 6). This would also make it possible to avoid the administration of potentially harmful antituberculous regimens.

## Concluding Remarks

This case report demonstrates that *K. kingae* might be considered as a potential cause of acute septic arthritis in children living in sub-Saharan Africa. Together with the evidence of *K. kingae* carriage among healthy children from Western Africa, these findings suggest that *K. kingae* might contribute to an underestimated burden of septic arthritis in this geographical area. Moreover, MLST analysis disclosed the first *K. kingae* STc in Africa that is a novel STc close to ST-26. Further prospective studies to specify the prevalence of *K. kingae* infection and carriage in sub-Saharan Africa are required to better help guiding rational diagnostic and therapeutic strategies.

## Consent for Publication

The written consent for publication was obtained from the parents’ child.

## Ethics Statement

The study was approved by the Ethics committee of the IHU Mediterranee-Infection under reference number 2016-024.

## Author Contributions

All the authors provided a substantial contribution to the conception and design of the work, and acquisition, analysis, and interpretation of data for the work. NEH and DC drafted the initial version of the manuscript, and all the authors revised it critically for important intellectual content. All the authors approved the present version to be published.

## Conflict of Interest Statement

The authors declare that the research was conducted in the absence of any commercial or financial relationships that could be construed as a potential conflict of interest.

## Abbreviations

CRP, C-reactive protein; DNA, deoxyribonucleic acid; MLST, multilocus sequence typing; PCR, polymerase chain reaction; ST, sequence type; STc, sequence-type complex.
