# Supplementary material for: Acute Septic Arthritis of the Knee Caused by Kingella kingae in a 5-Year-Old Cameroonian Boy
Source: Front Pediatr. 2017 Nov 6;5:230. doi: 10.3389/fped.2017.00230 (PMC5681518; doi:10.3389/fped.2017.00230)
Supplement: Supplementary file 1 [file presentation_1.pdf]

Supplementary Material

Acute septic arthritis of the knee caused by *Kingella kingae* in a 5-year-old Cameroonian boy

Nawal El Houmami[1*], Dimitri Ceroni[2], Karine Codjo-Seignon[1], Jean-Christophe Pons[1], Cédric Lambert[3], Guillaume André Durand[1], Philippe Minodier[4], Léopold Lamah[5], Philippe Bidet[6], Jacques Schrenzel[7], Didier Raoult[1], Pierre-Edouard Fournier[1]

*** Correspondence:** Dr. Nawal El Houmami: nawal.el-houmami@etu.univ-amu.fr or nawal.elho@gmail.com

1 Materials and Methods

Multicolour fluorescence *in situ* hybridization specific for *Kingella kingae*

Prior to apply multicolour fluorescence *in situ* hybridization assays on the synovial fluid from the patient, the method was validated with external positive and negative controls. Fresh cultures of the *Kingella kingae* KK199 and *K. negevensis* Sch538^T strains were used as positive control and negative controls, respectively. Bacterial colonies were suspended in phosphate-buffered saline, then formalin-fixed and paraffin-embedded. Thereafter, paraffin sections of 3,5 µm thick were stained simultaneously in red with the rhodamine-labeled probe bacterial EUB388 III (http://probebase.csb.univie.ac.at/pb_report/probe/161) that targets a consensus sequence of the bacterial 16S rRNA gene (**5'**– GCT GCC ACC CGT AGG TGT –**3'**), in green with a novel FITC-labeled probe designed to target specifically the *K. kingae* V1 region of the 16S rRNA gene (**5'**– CAC CAA GTA CAA GTA CTC GTG CTG –**3'**), and in blue using a DNA labeled DAPI probe. The red bacterial Cys5-labeled NONEUB probe (**5'**– ACT CCT ACG GGA GGC AGC –**3'**) was used as internal negative control (http://probebase.csb.univie.ac.at/pb_report/probe/243). Finally, a merged image was obtained by summing the four images corresponding to the four above-mentioned fluorophores.

2 Supplementary Figures and Tables

2.1 Figure S1

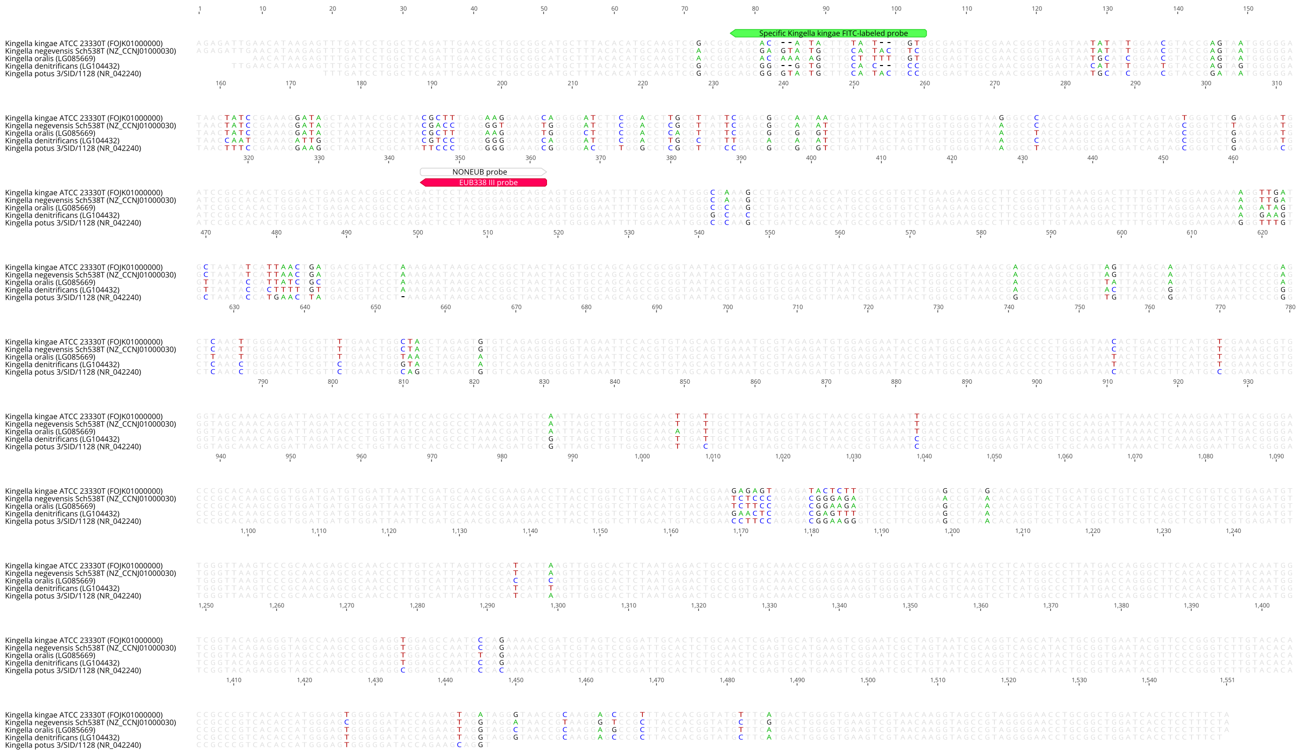

Figure S1. MAFFT alignment of the nucleotide sequences of the 16S rRNA gene from the five *Kingella* species, namely *K. kingae*, *K. negevensis*, *K. oralis*, *K. denitrificans*, and *K. potus*. Annotations indicate the position of the three probes targeting the 16S rRNA gene of *K. kingae* that were used in this study, namely the specific *K. kingae* FITC-labeled probe (green), the rhodamine-labeled EUB338 III probe (red), and the internal negative control Cys5-labeled NONEUB probe (white). MAFFT alignment was performed by using Geneious 10.2.3 (Biomatters).

2.2 Figure S2

Validation of the multicolour fluorescence *in situ* hybridization method to detect *K. kingae*

A. Positive control: *Kingella kingae* KK199

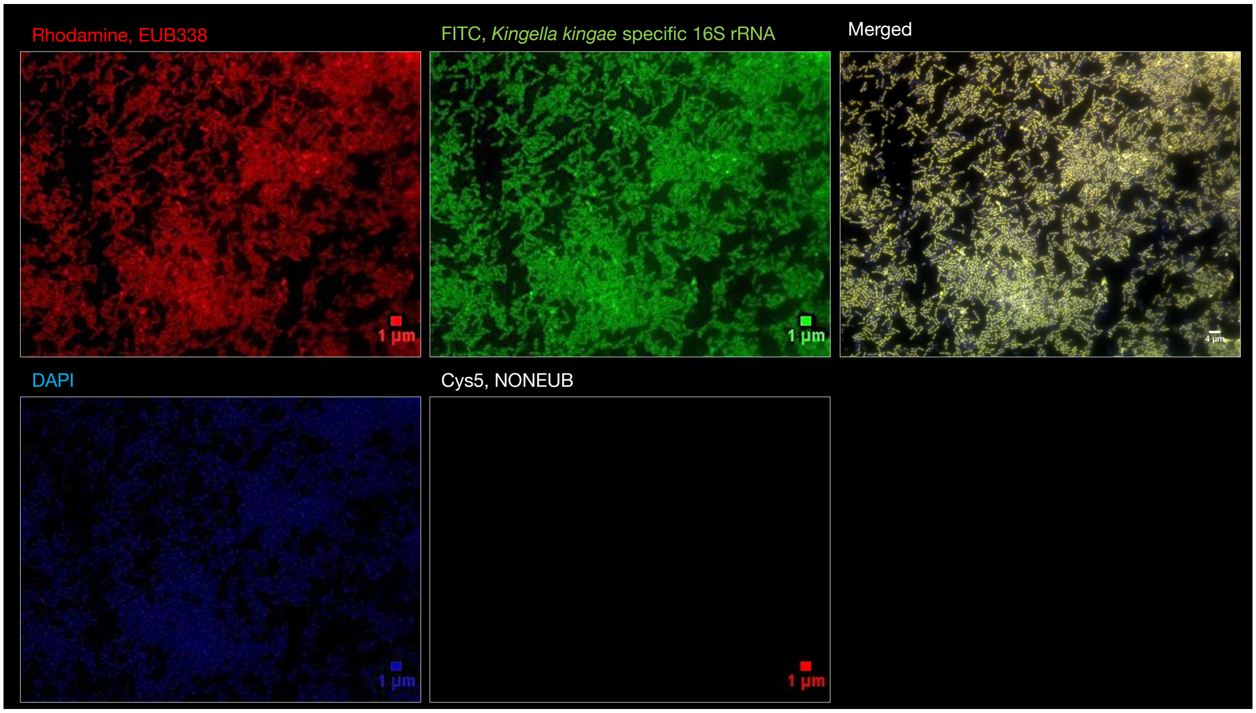

Figure S2A. *Kingella kingae* KK199 coccobacilli, which are arranged as pairs or short chains of cells, are visualised in red using the rhodamine-labeled probe bacterial EUB388 III (top left), in green using the specific *K. kingae* FITC-labeled probe (top middle), in blue using the DAPI probe (bottom left). As expected, no staining was obtained by using the bacterial Cys5-labeled NONEUB probe (bottom middle), which was used as internal negative control. The merged image (top right) was obtained by summing the four above-mentioned fluorophores.

B. Negative control: *Kingella negevensis* Sch538^{T}

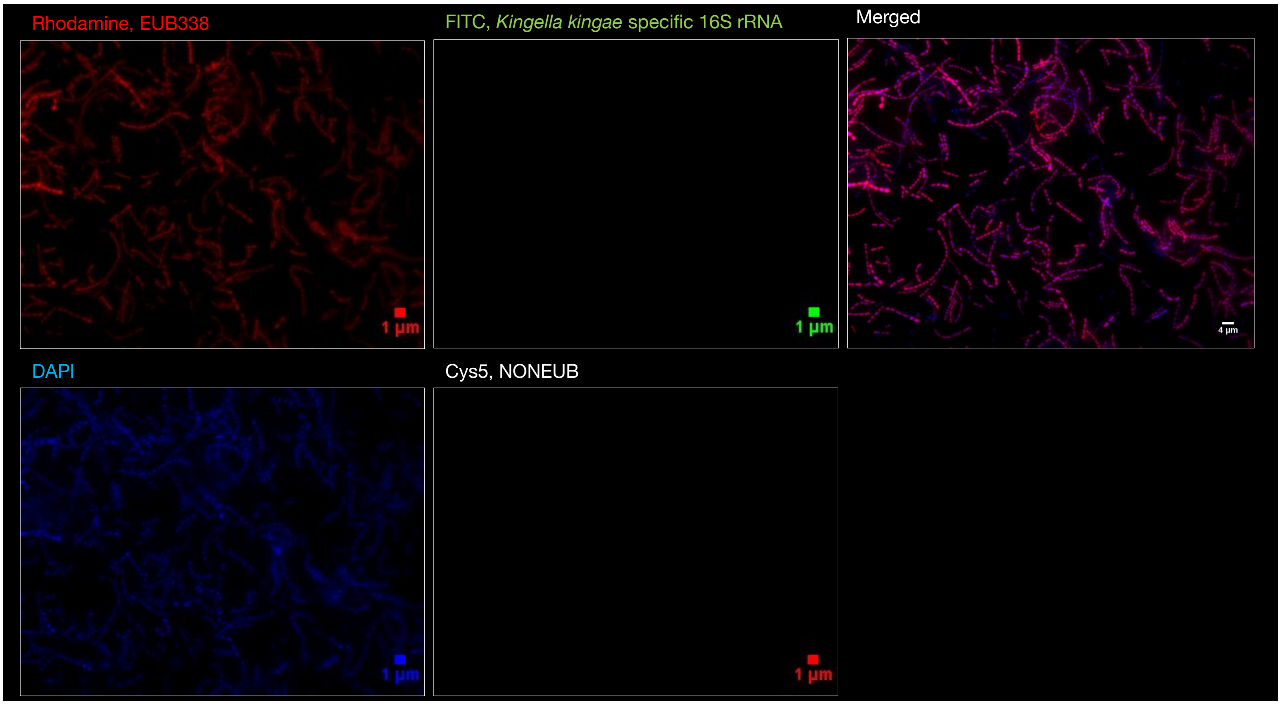

Figure S2B. *Kingella negevensis* Sch538^{T} cells, which are arranged in chains from 2 to 24 cells, are displayed in red using the rhodamine-labeled probe bacterial EUB388 III (top left) and in blue using the DAPI probe (bottom left). As expected, no staining was obtained by using the specific *K. kingae* FITC-labeled probe (top middle) and the bacterial Cys5-labeled NONEUB probe (bottom middle). The merged image (top right) was obtained by summing the four above-mentioned fluorophores.

2.3 Figure S3

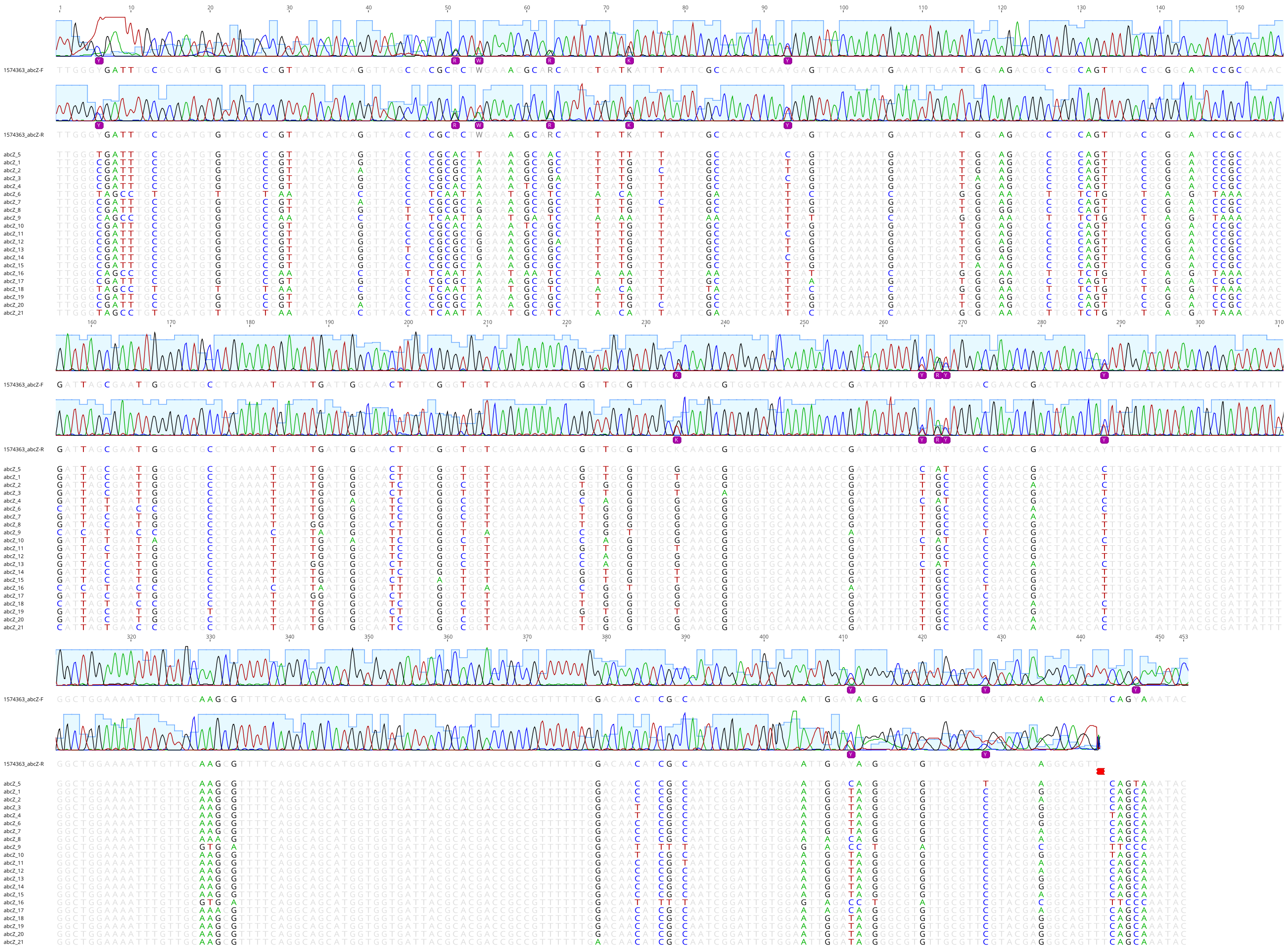

Figure S3. MAFFT alignment of nucleotide sequences of the *abcZ* alleles from the patient No. 1574363 (1574363_abcZ-F and 1574363_abcZ-R) with those registered in the MLST *Kingella kingae* database available by following this link (http://bigsdb.pasteur.fr/perl/bigsdb/bigsdb.pl?db=pubmlst_kingella_seqdef_public&page=downloadAlleles). The 14 ambiguous nucleotides identified are annotated in purple (Y [*n=8*]; R[*n=3*]; K[n=2]; W [*n=1*]). The MAFFT alignment was performed by using Geneious 10.2.3 (Biomatters).
